# A birth of bipartite exon by intragenic deletion

**DOI:** 10.1002/mgg3.277

**Published:** 2017-03-01

**Authors:** Kandai Nozu, Kazumoto Iijima, Toru Igarashi, Shiro Yamada, Jana Kralovicova, Yoshimi Nozu, Tomohiko Yamamura, Shogo Minamikawa, Ichiro Morioka, Takeshi Ninchoji, Hiroshi Kaito, Koichi Nakanishi, Igor Vorechovsky

**Affiliations:** ^1^ Department of PediatricsKobe University Graduate School of MedicineKobeJapan; ^2^ Department of PediatricsNippon Medical School HospitalTokyoJapan; ^3^ Department of PediatricsTokai University Oiso HospitalOisoJapan; ^4^ Division of Human GeneticsNational Institute of GeneticsMishimaJapan; ^5^ University of Southampton Faculty of MedicineSouthamptonUK; ^6^ Department of PediatricsWakayama Medical UniversityWakayamaJapan

**Keywords:** Alport syndrome, branch site, *COL4A5*, deletion, exon, LINE‐1, RNA, splicing, transposon

## Abstract

**Background:**

Disease‐causing mutations that activate transposon‐derived exons without creating a new splice‐site consensus have been reported rarely, but they provided unique insights into our understanding of structural motifs required for inclusion of intronic sequences in mature transcripts.

**Methods:**

We employ a combination of experimental and computational techniques to characterize the first de novo bipartite exon activation in genetic disease.

**Results:**

The exon originated from two separate introns as a result of an in‐frame *COL4A5* deletion associated with a typical Alport syndrome. The deletion encompassed exons 38 through 41 and activated a cryptic 3′ and 5′ splice site that were derived from intron 37 and intron 41, respectively. The deletion breakpoint was in the middle of the new exon, with considerable complementarity between the two exonic parts, potentially bringing the cryptic 3′ and 5′ splice site into proximity. The 3′ splice site, polypyrimidine tract and the branch site of the new exon were derived from an inactive, 5′ truncated LINE‐1 retrotransposon. This ancient LINE‐1 copy sustained a series of mutations that created the highly conserved AG dinucleotide at the 3′ splice site early in primate development. The exon was fully included in mature transcripts and introduced a stop codon in the shortened *COL4A5* mRNA, illustrating pitfalls of inferring disease severity from DNA mutation alone.

**Conclusion:**

These results expand the repertoire of mutational mechanisms that alter RNA processing in genetic disease and illustrate the extraordinary versatility of transposed elements in shaping the new exon‐intron structure and the phenotypic variability.

## Introduction

Hereditary diseases are often caused by intronic mutations that create new 3′ or 5′ splice sites to activate cryptic exons (Busslinger et al. 1981; Buratti et al. 2011). Mutation‐induced activation of cryptic splice sites is common in transposed elements, such as *Alu*s or mammalian‐wide interspersed repeats, contributing significantly to human morbidity, phenotypic variability, and the evolution of exon‐intron structure (Vorechovsky 2010; Schmitz and Brosius 2011). However, aberrant transcripts may also arise from intronic variants that alter auxiliary splicing sequences, known as enhancers and silencers (King et al. 2002; Pagani et al. 2002). Intronic splicing enhancers or silencers promote or inhibit recognition of cryptic splice sites, which have similar sequences as authentic splice sites but outnumber them by at least an order of magnitude in the genome (Fairbrother and Chasin 2000). Reports of disease‐causing mutations that activate new exons without creating a new splice‐site consensus have been sporadic, yet they have provided unique insights into our understanding of ancillary motifs required for inclusion of intronic sequences in mature transcripts (Ferlini et al. 1998; Pagani et al. 2002; Buratti et al. 2007b; Vorechovsky 2010; Kralovicova et al. 2015).

Alport syndrome is characterized by a progressive kidney disease accompanied by hearing loss and ocular abnormalities (Kashtan 1999). About 85% patients with Alport syndrome are due to mutations in the *COL4A5* gene (X‐linked Alport syndrome, XLAS, MIM:301050), which encodes the *α*5 chain of type IV collagen (Kashtan 1999). Over 900 different pathogenic *COL4A5* variants have been identified in XLAS, including large deletions, splice‐site mutations, and cryptic exon activation (King et al. 2002; Nozu et al. 2014a,b; Oka et al. 2014), but the XLAS mutation pattern is far from complete.

Here, we describe a heterozygous *COL4A5* deletion that activated a bipartite cryptic exon, with its 5′ and 3′ splice sites derived from distinct introns. The 3′ splice site, polypyrimidine tract and a branch site were contributed by an inactive copy of the Long INterspersed Element (LINE‐1), providing a new paradigm for the retrotransposon‐mediated phenotypic variability.

## Material and Methods

Genomic DNA was isolated from peripheral blood leukocytes of the proband and both parents using the Quick Gene Mini 80 System (Fujifilm, Tokyo, Japan) according to the manufacturer's instructions. Targeted next‐generation sequencing was carried out using the HaloPlex target enrichment system for the *COL4A3*,* COL4A4*, and *COL4A5* genes (Agilent Technologies, Santa Clara, California, USA) employing MiSeq. (Illumina, San Diego, California, USA) and SureCall (v. 3.0; Agilent Technologies). Multiplex Ligation‐dependent Probe Amplification (MLPA) was performed using the SALSA P191/P192 Alport assay (V.04; MRC‐Holland) according to supplier's recommendations. Total RNA was extracted from peripheral blood leukocytes with the Paxgene Blood RNA Kit (Qiagen, Venlo, The Netherlands) and reverse‐transcribed (RT) into complementary DNA (cDNA) using the Superscript III Kit (Invitrogen, Waltham, Massachusetts, USA). RT‐PCR primers were in exon 33/34 (gaa cct ggc tta cca ggt ata) and in exon 42 (agg acc ttc tgg acc tgg tag). A PCR product bridging the deletion breakpoint was amplified with primers in intron 37 (aag cac cac ata ttc aag ttt c) and intron 41 (aac ttg cat gtt aat tca gac c) from control and patient DNA. Direct sequencing of all PCR products was carried out as described (Nozu et al. 2014b).

RepeatMasker analyses were performed with the sensitive cross‐match search engine (v. 4) available at http://www.repeatmasker.org/. Prediction of RNA‐binding proteins that may contact consensus binding sites in the bipartite exon was carried out using RBPmap (Paz et al. 2014). RNA secondary structures were predicted with overlapping sequences encompassing the new exon using a free energy minimization algorithm implemented in RNAStructure (Mathews 2006). The intrinsic strength of cryptic splice sites activated by genomic deletion was estimated by computing their maximum entropy scores (Yeo and Burge 2004) and was compared with the mean scores previously established for authentic counterparts of mutation‐induced aberrant splice sites (Vorechovsky 2006; Buratti et al. 2007a). Auxiliary splicing sequences across the deletion breakpoint were examined against hexamer lists that were previously derived from computational and/or experimental studies of splicing enhancers or silencers (Fairbrother et al. 2004; Wang et al. 2004; Goren et al. 2006; Smith et al. 2006; Ke and Chasin 2010). The probability of unpaired (PU) values, which serve as a useful measure of single‐strandedness and correlate with functional splicing motifs, were computed as described (Hiller et al. 2007) using the new exon and 100 nucleotides (nt) of flanking intronic sequences as an input.

## Results

**Figure 1:**
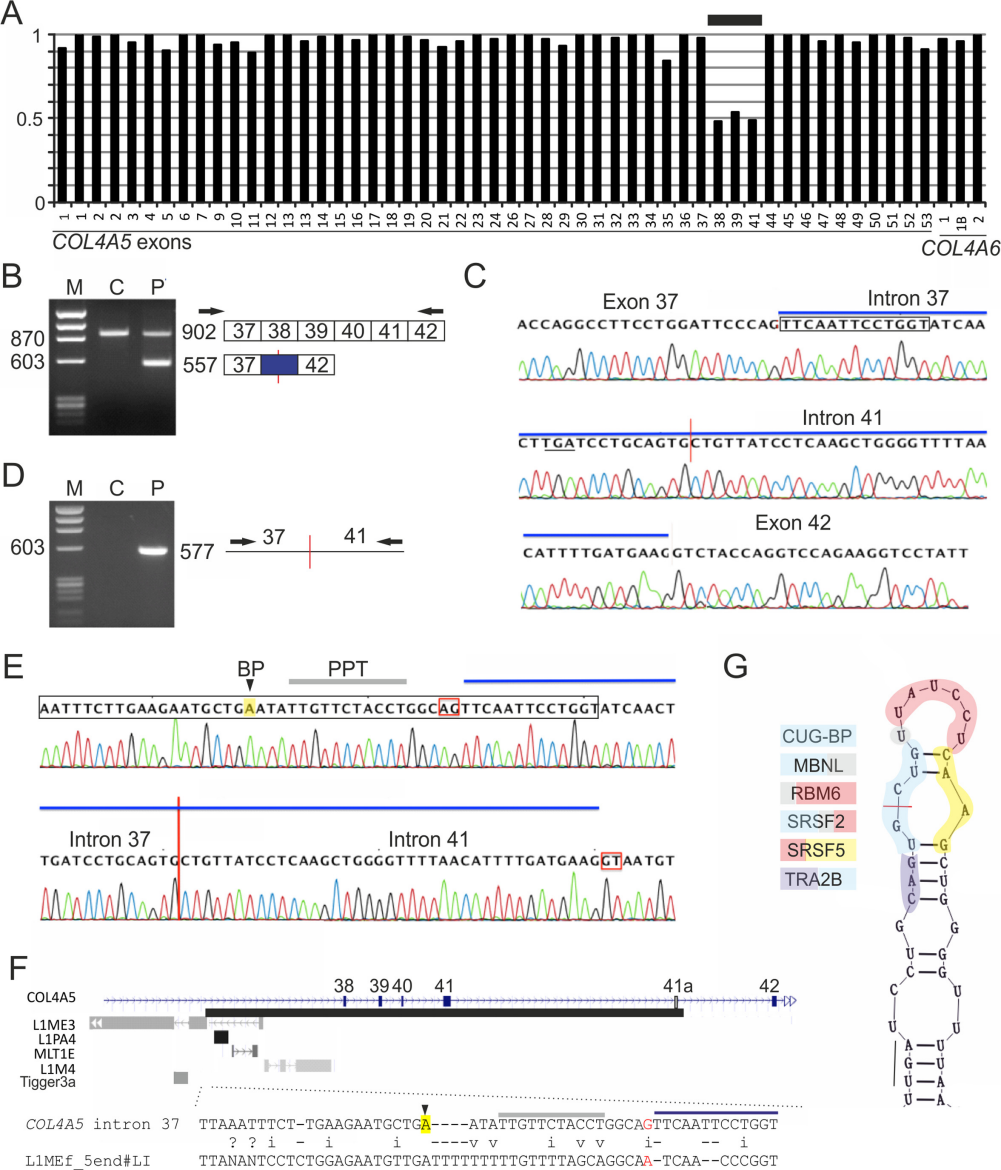
Genomic deletion activating a bipartite *COL4A5* exon in Alport syndrome. (A) Identification of a heterozygous deletion by multiplex ligation‐dependent probe amplification (MLPA) analysis of *COL4A5* exons. Exons with signal intensities at ~0.5 are denoted by a horizontal bar. *Y*‐axis, normalized MLPA values. Both parents showed a normal MLPA pattern and no evidence for mosaicism (data not shown). (B) RT‐PCR of control (C) and patient (P) total RNA samples. M, DNA size marker (nt). PCR products are shown schematically to the right. The new boundary is denoted by a vertical red line, the new exon is in blue and canonical exons are numbered. Amplification primers (arrows) were located in exon 33/34 and exon 42. (C) Sequence chromatogram of the aberrant cDNA product revealing a cryptic exon (blue bar). The L1 homology region (boxed) extends into the 5′ portion of the bipartite exon; stop codon is underlined. (D) PCR product amplified across the deletion breakpoint from control (C) and patient (P) DNA using primers in the indicated introns. (E) Sequence chromatogram of the corresponding fragment. For legend, see panel C. The polypyrimidine tract (PPT, gray bar) and the branch point adenine (BP, in yellow) of the new exon were predicted by a support vector machine (SVM) algorithm, with a SVM score of 0.81 (Corvelo et al. 2010). Conserved dinucleotides at new splice sites are in red boxes. (F) Summary of transposed elements across the centromeric deletion breakpoint (*upper panel*) and the alignment of the L1ME in *COL4A5* intron 37 with a L1ME consensus (*lower panel*). Mutation creating the AG dinucleotide in the *COL4A5 *L1ME is in red. (G) Putative interactions between RNA‐binding proteins and sequence motifs flanking the deletion breakpoint, as predicted by the RBPmap (Paz et al. 2014).

The proband was a 10‐year‐old girl without a family history of kidney disease. She was identified by chance hematuria and proteinuria. Her kidney biopsy showed characteristic basket‐weave changes of the glomerular basement membrane (Kashtan 1999) visualized by electron microscopy (data not shown), leading to the diagnosis of a typical Alport syndrome. However, next‐generation sequencing of *COL4A3*,* COL4A4*, and *COL4A5* with a HaloPlex target system failed to detect any pathogenic variants. The MLPA carried out as the next diagnostic step identified a heterozygous de novo deletion encompassing *COL4A5* exons 38 through 41 (Fig. 1A). Reverse transcriptase (RT)‐PCR followed by direct sequencing of cDNA showed that exons 38 to 41 were replaced by a 72‐nt insertion of a new exon, which was fully included in mature transcripts and introduced a stop codon in the mRNA (Fig. 1B,C). Direct sequencing of genomic DNA revealed that the new exon was bipartite, originating from intron 37 (33 nt) and intron 41 (39 nt), with deletion breakpoints at c.3373+6282 and c.3791‐2599 (Fig. 1D,E). The exon was surrounded by canonical AG and GT dinucleotides that characterize the vast majority of human introns (Fig. 1E).

**Figure 2:**
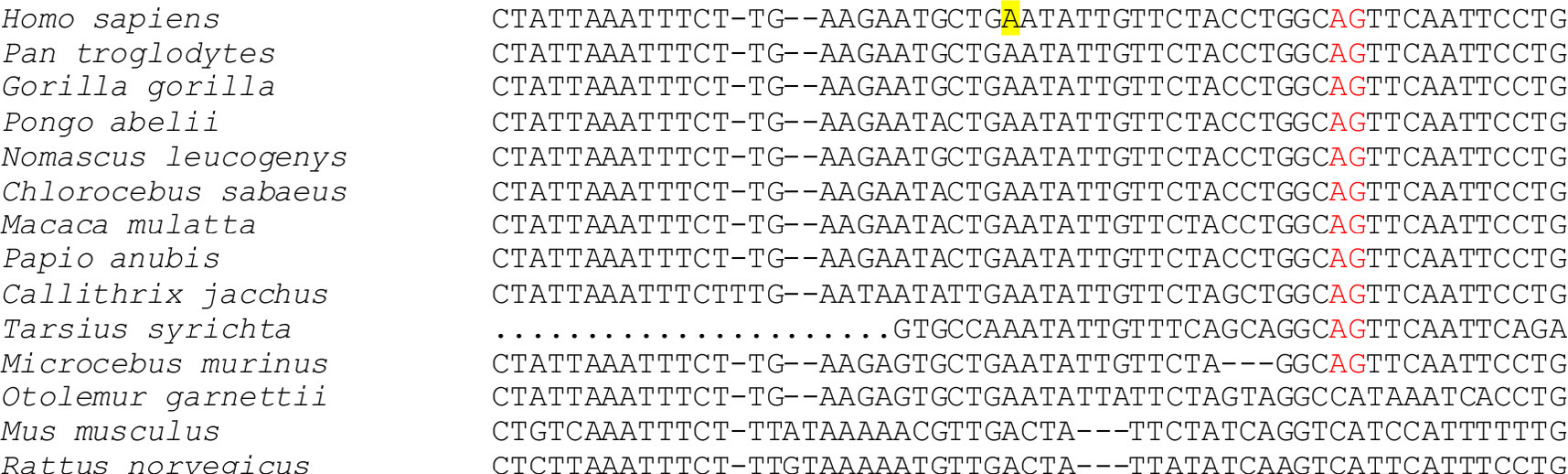
Sequence alignment of mammalian *COL4A5* orthologs across the L1 exon‐derived 3′ splice site**.** Conserved AG dinucleotide is in red, predicted branch point adenine in yellow. Alignment was created with full genomic reference sequences using Clustal Omega (v. 1.2.2). ‐, deletion, .., not determined.

RepeatMasker analysis of introns containing the deletion breakpoints revealed that the 3′ splice site and the 5′ part of the new exon were derived from an inactive antisense LINE‐1 (L1) element (Fig. 1F). This ancient L1ME copy also harbored the polypyrimidine tract and a high‐score branch site of the bipartite exon (Fig. 1E,F). The predicted branch point was located 20 nt upstream of the 3′ splice site, which was within the optimal distance previously estimated between 18 and 23 nt (Luukkonen and Séraphin 1997; Chua and Reed 2001). However, recognizable L1ME sequences did not extend into the 3′ portion of the intron 37‐derived exon segment, nor were any repetitive elements detected in the intron 41‐derived part of the new exon, including its 5′ splice site. Interestingly, a highly conserved AG dinucleotide at the 3′ splice site of the cryptic exon was absent in the L1ME consensus (Fig. 1F). Sequence alignments of mammalian *COL4A5* genes showed that the AG dinucleotide was absent in rodent L1 orthologs, but was present in all primates, except for *Otolemur garnettii* (Fig. 2). Assuming previously published estimates of the evolutionary age (Schmitz and Brosius 2011), this indicated that mutations required for the bipartite exon activation in our patient took place in primitive primates roughly 85 million years ago. Finally, comparison of the exonized *COL4A5* L1 repeat with the exon‐intron structure of ~150 existing human L1‐derived exons (Sela et al. 2007) failed to uncover any exons with a splice site at the same L1 position, revealing an entirely new type of deletion‐induced L1 exonization.

Accurate pre‐mRNA splicing depends on proper local folding of nascent transcripts, which can facilitate or inhibit splice‐site usage (Buratti et al. 2007b; Warf and Berglund 2010). Interestingly, secondary structure predictions of sequences surrounding the deletion breakpoint revealed significant complementarity between the end of shortened intron 37 and the remaining part of intron 41, which might facilitate formation of a stem that would bring the silent splice sites into proximity (Fig. S1). This structure could also support cross‐exon interactions that promote exon recognition, such as those involving serine/arginine‐rich proteins or other RNA‐binding factors predicted to bind motifs flanking the deletion breakpoint (Fig. 1G). The deletion created a new UGCU motif, which contributes to optimal binding sites of at least four splicing factors (Fig. 1G), including the well‐characterized YGCY site of muscle blind‐like proteins 1‐3 (MBNLs) (Taliaferro et al. 2016 and references therein). The minimal binding site of MBNLs (underlined above) was in a predicted single‐stranded conformation (Fig. 1G). The importance of RNA secondary structure in this exonization event was supported also by the absence of any predicted splicing enhancer hexamers across the deletion breakpoint (Table 1).

**Table 1:** Auxiliary splicing motifs created by the intronic fusion in *COL4A5*

New hexamer	Assignment (Ke and Chasin 2010)	PESE (Ke and Chasin 2010)	PESS (Ke and Chasin 2010)	RESCUE‐ESE (Fairbrother et al. 2004)	ESS (Wang et al. 2004)	ESR (Goren et al. 2006)	ESE (Smith et al. 2006)
CAGTGC	Silencer	‐	‐	‐	‐	‐	‐
AGTGCT	Silencer	‐	‐	‐	‐	+	‐
GTGCTG	Neutral	‐	‐	‐	‐	‐	‐
TGCTGT	Neutral	+	‐	‐	‐	‐	‐
GCTGTT	Neutral	+	‐	‐	‐	+	‐

The underlined portions of hexamer motifs are derived from intron 37, the remaining part from intron 41. Hexamers found in the motif list referenced at the top are denoted by a plus sign. Abbreviations for the auxiliary splicing motifs (columns 3–8) are explained in cited references.

**Figure 3:**
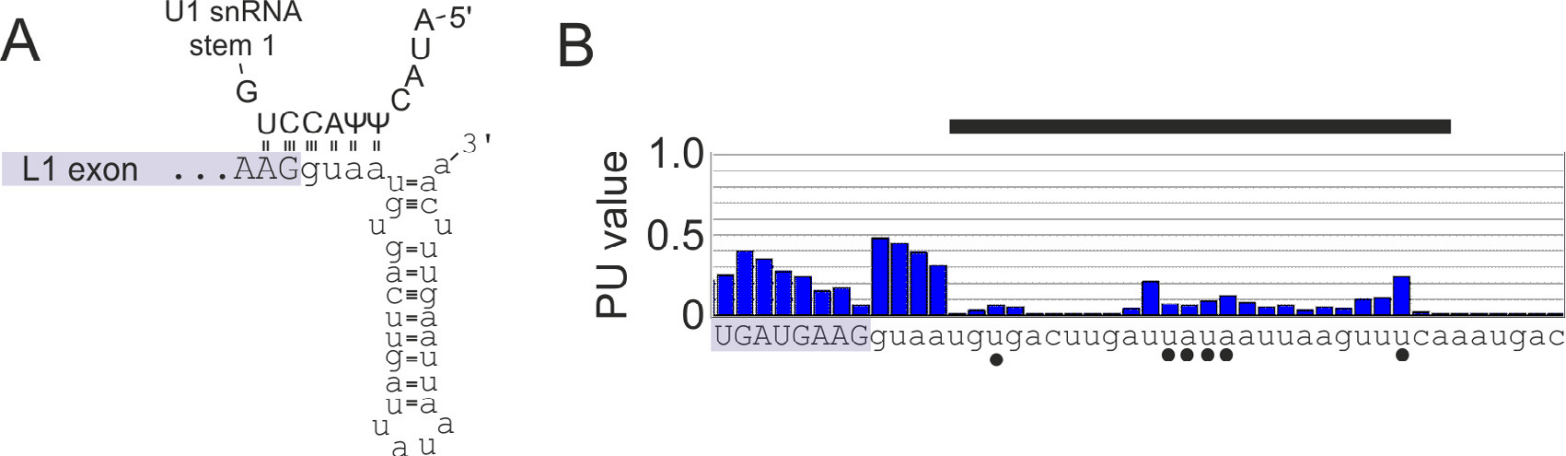
Predicted stem‐loop flanking the 5′ splice site of the bipartite exon. (A) Base‐pairing interactions between the U1 small nuclear RNA and the 5′ splice site. L1 exon (highlighted in purple) is in upper case, intron 41 sequences are in lower case, Ψ, pseudouridine. (B) PU values across this region. The predicted stem‐loop is denoted by a horizontal black bar and unpaired nucleotides of the stem‐loop by closed circles.

In contrast to auxiliary sequences, the intrinsic strength of both splice sites of the L1 exon was relatively high and was above the average for the 5′ splice site (Table 2). This splice site is flanked by a predicted stable helix, which extends up to the canonical base‐pairing between the U1 small nuclear RNA and 5′ splice site at core intron positions ‐2 through +4 (Fig. 3A). This interaction is probably stabilized by two pseudouridines at positions +3 and +4, which promote base‐stacking (Davis 1995). As the adjacent position +5 is usually occupied by a conserved guanine and its point mutations are particularly vulnerable to cryptic 5′ splice site activation (Buratti et al. 2007a), selection of the weaker, L1‐derived 3′ splice site may have been driven by the stronger 5′ splice site. Formation of the stable stem was supported by a very low probability of unpaired (PU) values computed across this region (Fig. 3B).

**Table 2:** The intrinsic strength of splice sites of the L1 exon in *COL4A5*

	Splice site	Maximum entropy score
*COL4A5* L1 exon	3′	6.1
	5′	9.0
Authentic splice sites	3′	7.9
	5′	7.6

## Discussion

To the best of our knowledge, this case represents the first de novo activation of a bipartite, “two‐intron” exon in genetic disease. This type of exonization has not been reported even for fusion transcripts that often arise in cancer cells as a result of genomic rearrangements (Professor Nick Cross, personal communication).

Importantly, without analyzing RNA products, the *COL4A5* deletion alone would be expected not to alter the reading frame (as in Ensembl transcript *COL4A5‐001*), omitting only three helix repeats from the entire collagen chain. In‐frame deletions have been associated with less severe ultrastructural kidney damage and/or XLAS phenotypes (Mazzucco et al. 1998; Nozu et al. 2014b), although dominant negative effects of the mutated allele cannot be excluded. Our results highlight the importance of characterizing aberrant transcripts for accurate prognosis of this patient and hereditary disorders in general, with potentially important implications for their management. Assuming that the nonsense transcript identified in this case (Fig. 1) is indeed a cause of the typical female XLAS, repression of the new L1 exon by mono‐ or bipartite splice‐switching oligonucleotides should increase the fraction of in‐frame transcripts and ameliorate the phenotype in a manner similar to antisense‐induced exon skipping in muscular dystrophy (Aartsma‐Rus 2010).

Symptomatic intragenic deletions often involve transposons (Guo et al. 2013) (Fig. 1F). L1s are the most abundant autonomous retrotransposons, with >0.5 million copies in the human genome (Lander et al. 2001). The majority of L1s are inactive, with only a hundred of full‐length copies capable of retrotransposition per genome (Brouha et al. 2003). *COL4A5* contains a single, potentially “hot” L1 in intron 1 (Mir et al. 2015), but the exonized L1ME copy has been inactive for a very long time as it is interrupted by younger elements, including a DNA transposon Tigger3 (Fig. 1F). The age of the primate‐specific Tigger3 family was estimated between 54 and 67 million years (Pace and Feschotte 2007).

As exemplified by the exonized *COL4A5*L1 (Fig. 1F), intronic L1 insertions are biased toward the antisense orientation relative to mRNAs, both in humans and mouse (Sela et al. 2007), possibly as a result of selective pressure acting to prevent interference between the L1 and the host gene transcription. However, LINEs do not exhibit preferential exonization orientation (Sela et al. 2007). Also, exonized sequences derived from L1 elements usually comprise the whole exon rather than only 3′ or 5′ splice site (Sela et al. 2007), suggesting that they can be recognized by the spliceosome without assistance from flanking unique sequences. In contrast, the L1 exonization in *COL4A5* was limited to the 5′ end of the repeat, with recognizable L1 sequences occupying only the 5′ part of the fusion exon (Fig. 1). Nevertheless, the overall exonization potential of intronic L1 repeats in evolution appears to be similar to other transposed elements (0.07%), but about 3× less than for *Alu*s (0.2%) (Sela et al. 2007), highlighting the unique character of the XLAS mutation.

Why were the cryptic splice sites normally separated by a long distance activated? RNA folding is an important modifier of exon selection, promoting or inhibiting splice‐site usage and binding affinities of numerous proteins in the spliceosome (Warf and Berglund 2010). Recent work suggested that local RNA structure limits rather than promotes binding of MBNL1 (Taliaferro et al. 2016), one of the candidate proteins that might bind new sequence motifs created by the deletion and (de‐)stabilize the stem‐loop (Fig. 1G). The predicted stable helix at the 5′ splice site could (de‐)repress splicing in the new genomic context (Fig. 3A). The helix is capped by an atypical UAUA tetraloop that was previously found in an RNase P ribozyme (Harris et al. 2001). Important precedents for this scenario include, for example, the 5′ splice sites of *SMN2* exon 7 and *MAPT* exon 10, which are regulated by the stability of adjacent stem‐loops (Donahue et al. 2006; Singh et al. 2007). The *MAPT* exon 10 hairpin interacts with the DDX5 helicase that promotes conformational change of the stem‐loop, increasing access to U1 (Kar et al. 2011). Finally, we cannot exclude that inclusion of the bipartite L1 exon in the *COL4A5* mRNA is immune to the deletion‐mediated disruption in the transcription elongation rate (Han et al. 2004), which could affect RNA polymerase II processivity, RNA folding and kinetics of the spliceosome assembly (Luco et al. 2011 and references therein). Identification of key RNA‐RNA and RNA‐protein interactions that promoted the birth of the cryptic L1 exon will be required to test these hypotheses in the future studies.

In conclusion, our case report demonstrates the astonishing versatility of intragenic deletions and transposed elements in shaping the new exon‐intron structure, expanding the repertoire of currently known L1‐mediated morbidities (Narita et al. 1993; Chen et al. 2005; Vorechovsky 2010). Our XLAS case also highlights the perilous inadequacy of predicting phenotypic severity of Mendelian disorders from DNA changes alone. As with exonized mammalian‐wide interspersed repeats (Kralovicova et al. 2015), future systematic analyses of L1‐derived 3′ splice sites should help characterize RNA interactions that facilitate their recognition by the spliceosome. Finally, our results suggest that the fraction of disease‐causing intragenic deletions that affect RNA processing could be much larger than anticipated and that such cases may provide valuable exon selection models for future studies.

### Ethical compliance

All procedures were reviewed and approved by the Institutional Review Board of Kobe University School of Medicine. Informed consent was obtained from proband's parents.

## Conflict of Interest

The authors have nothing to disclose.

## Supporting information


**Figure S1.** Complementarity of the 5′ and 3′ parts of the bipartite exon.
