# Supplementary material for: A birth of bipartite exon by intragenic deletion
Source: Mol Genet Genomic Med. 2017 Mar 1;5(3):287–94. doi: 10.1002/mgg3.277 (PMC5441408; doi:10.1002/mgg3.277)

Figure S1

Reverse complementarity of 5' and 3' parts of the bipartite exon

Legend: Location of splice sites (blue lines) and the deletion breakpoint (red line) in the most stable secondary structure across the L1 exon, as predicted by RNAStructure (v. 5.8). The L1-derived segment is denoted by a black line.

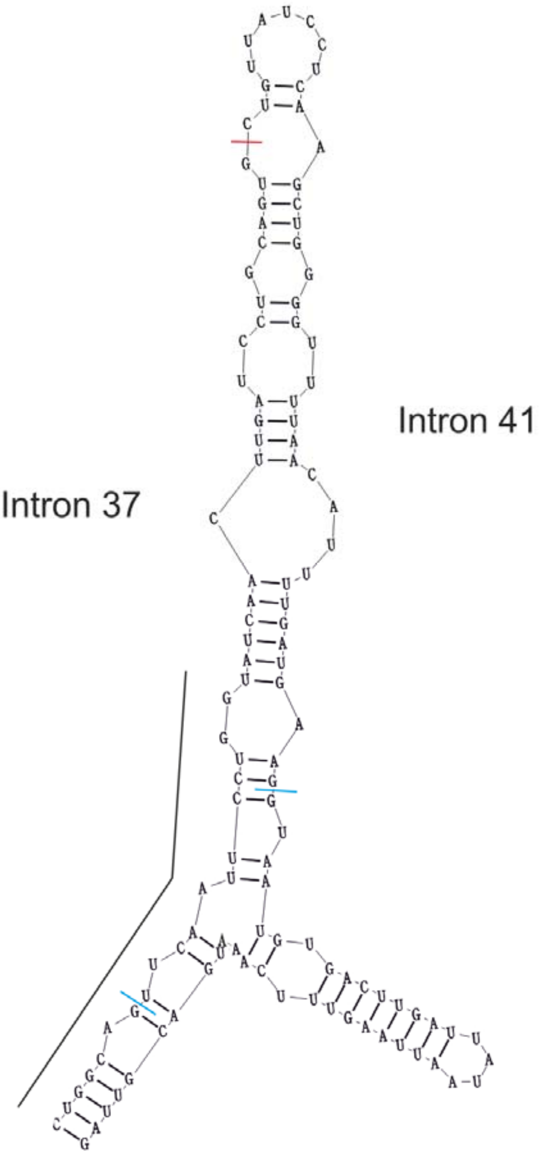

Supplement: Supplementary file 1 — Figure S1. Complementarity of the 5′ and 3′ parts of the bipartite exon. [file MGG3-5-287-s001.pdf]
